# Psychosocial Distress and Radiation Treatment Interruptions in Patients With Cancer Using Machine Learning and Regression: Retrospective Cohort Study

**DOI:** 10.2196/92899

**Published:** 2026-10-19

**Authors:** Soheil Hashtarkhani, Christopher L Brett, Fekede Asefa Kumsa, Rezaur Rashid, Parnian Kheirkhah Rahimabad, Saba Kheirinejad, Robert L Davis, David L Schwartz, Arash Shaban-Nejad

**Affiliations:** 1 Center for Biomedical Informatics, College of Medicine, University of Tennessee Health Science Center, 50 N Dunlap Street, Memphis, TN, 38103, United States, 1 9012875836; 2 Health Equity Program, Rhodes College, Memphis, TN, United States; 3 University of Tennessee Graduate School of Medicine, Knoxville, TN, United States; 4 Department of Radiation Oncology, University of Tennessee Health Science Center, College of Medicine, Memphis, TN, United States; 5 Department of Preventive Medicine, University of Tennessee Health Science Center, College of Medicine, Memphis, TN, United States

**Keywords:** radiotherapy, cancer, treatment interruption, risk factors, psychosocial distress, machine learning, extreme gradient boosting, XGBoost, negative binomial regression

## Abstract

**Background:**

Uninterrupted delivery of radiation therapy is critical to cancer treatment quality. Unplanned radiation treatment interruptions compromise tumor control and survival. Although previous studies have explored clinical and sociodemographic factors influencing treatment adherence, the role of psychosocial distress and related symptoms remains unclear.

**Objective:**

This study aims to identify patient-reported psychosocial predictors of radiation treatment interruptions from patient-reported distress screening data integrated with clinical and socioeconomic correlates via traditional and machine learning–enhanced modeling.

**Methods:**

This institutional cohort study included 1953 patients who underwent external beam radiation therapy between August 2020 and May 2024. Patient demographics, clinical characteristics, treatment details, and baseline distress levels were extracted from a hospital-level electronic health record data repository. Distress was assessed using the Distress Thermometer developed by the National Comprehensive Cancer Network. These data were linked with census tract–level socioeconomic indicators. Predictors of treatment interruption (measured as missed treatment days) were evaluated using negative binomial regression and an extreme gradient boosting (XGBoost) model with a Poisson objective function.

**Results:**

The study cohort missed a mean of 1.19 (SD 2.18) treatment days (range: 0-15). Higher distress scores, the presence of learning or communication barriers, and genitourinary malignancy were significantly associated with increased interruption risk in the negative binomial regression model. The XGBoost model confirmed these features as top contributors to prediction performance, while yielding an incrementally lower root mean squared error of 2.19 (vs 2.21 for the regression model).

**Conclusions:**

Patient-reported distress and health education barriers were modestly associated with treatment interruptions, underscoring the importance of incorporating psychosocial assessments into radiation oncology workflows. Both parametric and machine learning models provided stable and closely aligned predictions of missed radiation treatment days. Key predictors identified as statistically significant in the regression model were also among the top contributors in the XGBoost model, reinforcing the robustness of these findings and supporting integration of both approaches into future intervention strategies.

## Introduction

Radiation therapy is a fundamental component of cancer management, with more than half of all patients requiring radiotherapy during their treatment course [1]. The effectiveness of radiation therapy depends on consistent treatment delivery, as unplanned interruptions have been associated with reduced tumor control, disease recurrence, and decreased overall survival [2,3]. Despite ongoing technological and procedural advances, radiation treatment interruptions (RTIs) remain a major challenge in oncology care. These interruptions arise from multiple factors, including technical and operational issues [4,5], as well as patient-specific factors such as poor performance status, treatment-related toxicities, and unplanned hospitalizations [6]. Social determinants of health further compound these challenges, as individuals with limited financial means, lower education, or unstable support systems are more likely to experience barriers to treatment completion [7].

While previous studies have identified several demographic, clinical, and socioeconomic factors associated with RTIs [8-10], less is known about how psychological and symptom-related domains interact with these traditional predictors to influence treatment adherence. Cancer-related distress, encompassing emotional, physical, and practical challenges, is a prevalent yet often underrecognized factor that may directly contribute to treatment noncompliance [11,12]. Patients experiencing high levels of distress or other psychosocial difficulties may have challenges maintaining daily treatment schedules due to fatigue, anxiety, or logistical stressors, leading to preventable treatment delays. We integrated regression-based analyses with machine learning methods to examine the influence of psychological and distress-related symptoms, along with other key variables, on RTIs.

The present study aims to comprehensively evaluate the predictors of RTIs within a Central Appalachian micropolitan and rural catchment region. By integrating patient-reported distress screening data with clinical and socioeconomic information, this analysis sought to capture the multidimensional influences on treatment adherence. The overarching goal was to identify key risk factors that may inform targeted interventions, improve continuity of care, and ultimately enhance the quality and equity of radiation oncology delivery in underserved and geographically diverse communities.

## Methods

### Dataset

Demographic, clinical, and treatment-related data, including age, sex, race, cancer site, insurance type, and marital status, were obtained from the centralized oncology data repository at the University of Tennessee Medical Center in Knoxville, Tennessee. The analytic cohort included patients who received external beam radiation therapy between August 2020 and May 2024. This cohort draws from the same institutional data source as our group’s prior analysis of demographic and clinical predictors of treatment interruption [9]; the present study extends that work by incorporating patient-reported psychosocial distress screening data and census tract–level socioeconomic indicators, which were not examined previously.

Patients undergoing treatment were asked to complete a standardized distress screening tool at baseline before starting radiation therapy. This instrument canvassed emotional, physical, practical, and nutritional concerns, along with a self-reported distress score ranging from 0 (“no distress”) to 10 (“extreme distress”). Paper-based forms were reviewed by nursing staff with the patient, with the distress score and pertinent findings entered into an electronic form within the medical record, enabling integration of psychological and symptom-related measures with clinical and demographic records.

To complement patient-level data, area-level social determinants of health were appended using data from the US Census Bureau’s American Community Survey 2019‐2023 [13], aggregated at the census tract level. Socioeconomic indicators included measures such as median household income and educational attainment in patients’ home census tracts, enabling contextual assessment of neighborhood characteristics.

### Preprocessing and Cohort Selection

A total of 3134 consecutive patients underwent radiation therapy during the study period. Several data-quality and eligibility criteria were applied to define the analytic cohort. First, 269 patients were excluded for missing more than fifteen treatment days at the data extraction stage. Interruptions of this length were considered unlikely to represent a single episodic treatment interruption and more often reflect distinct clinical scenarios, such as sequential courses of radiotherapy for different disease sites; consequently, detailed records for these patients were not retained for further characterization. Patients receiving treatment courses lasting fewer than 5 days (n=599) or with a total delivered dose below 2000 cGy (n=84) were also removed, as these treatment patterns generally correspond to very short palliative or stereotactic regimens in which the clinical significance of interruptions differs from that of conventional fractionation. Additional exclusions were made for missing demographic or administrative variables (52 cases). These steps resulted in 2130 patients with complete information. Among these individuals, 177 patients did not complete a baseline distress screening form and could not be included in analyses incorporating psychological variables, resulting in a final analytic sample of 1953.

Categorical variables were harmonized and standardized, with missing categories explicitly coded to maintain transparency in downstream modeling. All continuous variables and clinical indicators were cleaned using context-specific rules to preserve interpretability. Socioeconomic and demographic measures were grouped into broader categories to reduce data sparsity.

Multicollinearity among predictors was evaluated using the variance inflation factor (VIF) and generalized VIF (GVIF) for categorical predictors with multiple levels. All predictors exhibited acceptable collinearity levels (GVIF-adjusted statistics <10, most <2.5), indicating no significant multicollinearity concerns. Therefore, all candidate variables were retained for inclusion in subsequent modeling, including both negative binomial regression and extreme gradient boosting (XGBoost) analyses.

### Study Outcome Measures

The primary outcome of this study was the number of missed radiation treatment days. Missed treatment days were calculated based on the anticipated versus actual completion dates of therapy, adjusted for weekends and federal holidays. This count variable, which fell within a 0 to 15 day range, represents the total number of treatment days not completed as scheduled.

### Automated Variable Categorization Using Large Language Models

Several extracted variables were available only in unstructured text format and required additional processing before they could be used for analysis. For example, *International Classification of Diseases* (*ICD*) diagnosis codes and insurance providers needed to be mapped into standardized categorical groups. In addition, approximately 7% of the distress screening forms included free-text entries by nursing staff rather than the designated radio buttons for practical, emotional, physical, and nutritional concerns.

To address this, we incorporated GPT-4o APIs into the preprocessing pipeline. GPT-4o was selected based on our group’s prior comparative evaluation of 5 language models (GPT-3.5, GPT-4o, Llama 3.2, Gemini 1.5, and BioBERT) for clinical text categorization tasks in an oncology setting, in which GPT-4o and BioBERT outperformed the other models [14]. The present study required categorization of a structured variable (*International Classification of Diseases*, 10th Revision [*ICD*-10], with over 50 unique values), a semistructured variable (insurance, with over 700 unique values), and an unstructured variable (free-text distress screening entries).

The categorization framework and mapping schema were developed in consultation with 2 radiation oncology specialists. A few-shot prompting strategy was used, in which the model was provided with a small set of representative examples to guide consistent categorization for each variable type. LLM-generated categorizations were subsequently reviewed by our 2 oncology coauthors, allowing us to both correct any misclassifications and assess the feasibility of using LLM-based categorization independently, without manual review, in future prediction pipelines.

### Model Generation

Missed radiation treatment day values were heavily right-skewed, with a large proportion of zero counts and a long upper tail. This distribution confirmed that the data were discrete and overdispersed, warranting the use of count-based regression rather than continuous linear models.

The dataset was divided into training (391/1953, 80%) and testing (391/1953, 20%) subsets, with categorical variables one-hot encoded for the machine learning algorithm. To assess the stability of performance estimates given this single split, we additionally conducted 100 repeated stratified resamples preserving the distribution of missed treatment days across train and test sets, and calculated mean, SD, and 95% CIs (2.5th-97.5th percentile) for root mean square error (RMSE), mean absolute error (MAE), and Poisson deviance across resamples.

A negative binomial regression model was fitted to the training data to account for overdispersion, as the initial Poisson model exhibited a dispersion statistic of 3.69. The negative binomial model estimated incidence rate ratios (IRRs) for each covariate, providing interpretable measures of relative change in missed treatment days associated with each factor. To ensure model parsimony while preserving interpretability, the full negative binomial model including all candidate covariates was first examined. Backward elimination was then applied as a sensitivity approach to remove nonsignificant predictors (*P*≥.05); this process did not materially alter effect estimates or the substantive conclusions of the model. Given the large proportion of zero outcomes, we additionally evaluated a zero-inflated negative binomial model as part of model selection; however, the zero-inflation component was not statistically significant, predictive performance on the hold-out test set did not improve, and a Vuong test favored the standard negative binomial formulation. Therefore, the conventional negative binomial model was retained for all primary analyses.

In parallel, an XGBoost model was trained on the same training data using a Poisson objective function to handle count-based outcomes. Hyperparameters, including tree depth, learning rate, subsampling rate, and column sampling, were tuned through internal validation and early stopping to prevent overfitting. Both models were subsequently applied to the test dataset to generate predicted counts and assess generalization performance.

### Model Comparison

Predictive accuracy was assessed using multiple complementary metrics—RMSE, MAE, and Poisson deviance—to capture different aspects of model fit and prediction error. All performance metrics were calculated on a naive 20% hold-out test set, which was separated from the data prior to model training or hyperparameter tuning to ensure an unbiased evaluation of out-of-sample performance. In addition to evaluating predictive performance, a direct comparison of these two modeling approaches enabled integration of statistical inference and machine learning–based feature importance.

### Ethical Considerations

This study was reviewed and approved by the Institutional Review Board of the University of Tennessee Health Science Center for analysis without personal identifiers (protocol: 16‐04888-XP).

## Results

### Descriptive Analysis

Table 1 presents the demographic, socioeconomic, and clinical characteristics of the study cohort. The total number of RTIs ranged from 0 to 15 days, with a mean of 1.19 (SD 2.18) days (median 0, IQR 0‐1). Study patients had a mean age of 64.5 (SD 12.1) years and were predominantly White (1837/1953, 94.1%), with smaller proportions identifying as Black/African American (88/1953, 4.5%) or other/minority (28/1953, 1.4%). The cohort included 1017 (52.1%) females and most participants were married (1130/1953, 57.9%) and received health insurance coverage through Medicare (647/1953, 33.1%) or commercial payers (776/1953, 39.7%). Approximately one-third of patients resided in low-income (596/1953, 30.5%) or low-education (589/1953, 30.2%) areas.

**Table 1.** Characteristics of the study cohort (N=1953).

Variable	Description	Range/coding	Distribution
Outcome			
Missing treatment days (outcome)	Number of missed radiation treatment sessions compared to prescribed schedule	0‐15 days	Mean 1.19 (SD 2.18) Median 0 (IQR 0‐1)
Distress and symptom measures			
Distress score	Self-reported oncology distress thermometer score assessing psychological distress during treatment	0‐10	Mean 3.1 (SD 2.9) Median 3 (IQR 0-5; range 0‐10)
Emotional symptom count	Number of emotional or psychological concerns selected (eg, depression, sadness, fears, and nervousness)	0=“no concern” and 1‐6=“number of emotional issues”	0: 1769 (90.6%) 1: 66 (3.4%) 2: 52 (2.7%) 3: 19 (1.0%) 4: 15 (0.8%) 5: 16 (0.8%) 6: 16 (0.8%)
Practical symptom count	Number of practical or logistical concerns endorsed (eg, financial, transportation, and insurance issues)	0=“none” and 1‐5=“number of problems”	0: 1855 (95.0%) 1: 54 (2.8%) 2: 31 (1.6%) 3: 5 (0.3%) 4: 3 (0.2%) 5: 5 (0.3%)
Physical symptom count	Number of physical concerns (eg, fatigue, sleep disturbance, and mobility problems)	0‐13	0: 1738 (89.0%) 1: 85 (4.4%) 2: 29 (1.5%) 3: 21 (1.1%) 4: 23 (1.2%) ≥5: 57 (2.9%)
Nutrition symptom count	Number of nutrition-related problems (eg, nausea, appetite loss, and diarrhea)	0‐5	0: 1847 (94.6%) 1: 64 (3.3%) 2: 25 (1.3%) 3: 13 (0.7%) ≥4: 5 (0.3%)
Primary pain intensity	Self-reported primary pain score at assessment	0‐10 scale	0: 1435 (73.5%) 1-3: 195 (10.0%) 4-6: 172 (8.8%) 7-10: 151 (7.7%)
Fall risk and clinical measures			
Fall risk (medication)	Risk level based on use of medications contributing to falls	No risk, polypharmacy, and high-risk medications	No risk: 964 (49.4%) Polypharmacy: 805 (41.2%) High-risk medications: 184 (9.4%)
Fall risk (elimination)	Risk based on urinary or bowel elimination issues	Independent and at risk	Independent: 1919 (98.3%) At risk: 34 (1.7%)
Fall history	Any history of prior falls	Yes/no	Yes: 80 (4.1%) No: 1873 (95.9%)
Falls assessment score	Composite nursing assessment fall-risk score	0‐9	Median 1 (IQR 0‐2) 0: 595 (30.5%) 1: 630 (32.3%) 2: 444 (22.7%) ≥3: 284 (14.5%)
Falls risk band	Categorized fall risk level derived from score	Low/moderate/high	Low (62.8%) Moderate (27.1%) High (10.1%)
ECOG^a^ performance status	Clinical performance status scale (0‐4)	0=“Fully active”; 4=“Completely disabled”	0: 1505 (77.1%) 1: 371 (19.0%) 2: 51 (2.6%) 3: 26 (1.3%)
Has barrier to learning	Presence of nurse-identified communication or learning barriers during care	0=“No”; 1=“Yes”	No: 1662 (85.1%) Yes: 291 (14.9%)
Influenza vaccine	Record of influenza vaccination	Yes/no	Yes: 769 (39.4%) No: 1184 (60.6%)
Fears or anxieties	Presence of fears or anxiety-related symptoms	Yes/no	Yes: 259 (13.3%) No: 1694 (86.7%)
Living will/advance care plan	Documented advance care planning	Yes/no	Yes: 499 (25.6%) No: 1454 (74.4%)
Demographic and socioeconomic variables			
Age	Age in years at the start of radiation therapy	Continuous variable	Mean 64.5 (SD 12.1; range 21‐95)
Race	Self-identified race	White, Black/African American, and other	White: 1837 (94.1%) Black: 88 (4.5%) Other: 28 (1.4%)
Sex	Self-identified sex	Female and male	Female: 1017 (52.1%) Male: 936 (47.9%)
Marital status	Marital status at diagnosis	Married, divorced, single, widowed, and other	Married: 1130 (57.9%) Single: 312 (16.0%) Divorced: 176 (9.0%) Widowed: 159 (8.1%) Other: 176 (9.0%)
*ICD*-10^b^ category^c^ (cancer site)	Primary cancer site category	14 *ICD*-10–based groups	Metastasis: 395 (20.2%) Breast: 315 (16.1%) Prostate: 221 (11.3%) Head/neck: 133 (6.8%)
Insurance category	Primary insurance type at treatment	Medicare, Medicaid, commercial, and so on	Medicare: 647 (33.1%) Commercial: 776 (39.7%) Medicaid: 112 (5.7%) Dual: 61 (3.1%) Other: 168 (8.6%) Missing category
Median household income	Area-level median income group	Low, moderate, and high	Low: 596 (30.5%) Moderate: 649 (33.2%) High: 707 (36.2%)
Percent population ≥ bachelor’s degree	Educational attainment level of census area	Low, moderate, and high	Low: 589 (30.2%) Moderate: 651 (33.3%) High: 713 (36.5%)
Smoking status	Smoking history	Current, former, never, and unknown	Current: 183 (9.4%) Former: 355 (18.2%) Never: 430 (22.0%) Unknown: 985 (50.4%)
Alcohol use	Self-reported alcohol use	Current, past, denies, and unknown	Denies: 628 (32.2%) Current: 463 (23.7%) Past: 110 (5.6%) Unknown: 752 (38.5%)
Distance to radiation facility	Straight-line distance to treating radiation center (miles)	Continuous variable	Median 13.5 (IQR 7.8‐25.1; range: 0.9‐255) miles

a ECOG: Eastern cooperative oncology group.

b *ICD*-10: *International Classification of Diseases*, 10th Revision.

c The 4 most common of 14 groups are shown.

The mean distress score was 3.1 (SD 2.9), with a range of 0‐10 indicating generally moderate distress with considerable variability across the cohort. Emotional, physical, practical, and nutritional symptom variables represent the number of symptom categories patients endorsed within each domain. For emotional symptoms, 90.6% (n=1769) reported no emotional concerns, 3.4% (n=66) endorsed one emotional symptom, and fewer than 3% (n≤52) of patients in each remaining category endorsed two or more emotional symptoms. The majority of patients demonstrated good functional status, with 96% (1876/1953) scoring 0‐1 on the Eastern Cooperative Oncology Group (ECOG) performance scale and exhibited low to moderate fall risk. The median travel distance from home address to the radiation treatment facility was 13.5 (IQR 7.8‐25.1) miles.

A total of 177 patients were excluded from the analytic cohort due to missing distress score values. To assess whether their exclusion introduced systematic bias, we compared key demographic characteristics with those of the included cohort. The excluded patients were similar in age (median 64, IQR 56.8-73.7 y vs median 64.5, IQR 57.6-72.9 y), sex distribution (female: 102/177, 57.7% vs 1017/1953, 52.1%), and race (White: 167/177, 94.3% vs 1838/1953, 94.1%). Marital status and insurance patterns also aligned closely with the main study population, indicating no meaningful demographic differences. Excluded patients also had a similar rate of missed treatment days (mean 1.09, SD 1.96; 67/177, 38.1% with any interruption) compared with included patients (mean 1.19, SD 2.18; 842/1953, 43.1% with any interruption), with no significant difference between groups by Mann-Whitney *U* test (*P*=.41), indicating no meaningful demographic or outcome-related differences between groups.

### Negative Binomial Regression Modeling

Negative binomial regression identified multiple variables significantly associated with treatment interruptions (Table 2). Among psychosocial and clinical variables, patients’ barriers to health-related communication or learning during care were associated with a 43% higher rate of missed sessions (IRR 1.43, 95% CI 1.07‐1.92; *P*=.02). Each one-point increase in total distress score corresponded to a 5% higher rate of missed treatments (IRR 1.05, 95% CI 1.02‐1.09; *P*=.01).

**Table 2.** Negative binomial regression model results.

Variable	IRR^a^ (95% CI)	*P* value
*ICD*-10^b^: genitourinary	2.57 (1.33‐4.97)	.006
Distress score (per unit)	1.05 (1.02‐1.09)	.01
Marital status: married	0.67 (0.49‐0.92)	.02
Learning barrier (yes vs no)	1.43 (1.07‐1.92)	.02
Physical symptom count	0.82 (0.72‐0.96)	.02
Nutrition symptom count	1.43 (0.99‐2.11)	.051
*ICD*-10: prostate	1.52 (0.99‐2.32)	.053
Marital status: widowed	0.65 (0.42‐1.02)	.06
Race: White vs others	0.69 (0.46‐1.02)	.06
Insurance: other insurance	0.37 (0.11‐1.06)	.07
Distance to facility (per mile)	1.00 (0.99‐1.00)	.08
Marital status: single	0.76 (0.53‐1.09)	.13
Age (per y)	0.99 (0.98‐1.00)	.14
*ICD*-10: head and neck	1.42 (0.87‐2.31)	.16
Fall risk (elimination; independent vs at risk)	1.82 (0.78‐4.18)	.17
Living will/advance care (yes vs no)	1.16 (0.93‐1.43)	.18
*ICD*-10: skin	1.40 (0.81‐2.43)	.22
ECOG^c^ performance	1.10 (0.91‐1.33)	.30
Primary pain intensity	1.02 (0.98‐1.06)	.31
Influenza vaccine (yes vs no)	0.91 (0.75‐1.11)	.35
*ICD*-10: other	1.31 (0.74‐2.34)	.35
Falls assessment score	1.07 (0.91‐1.27)	.37
Emotional symptom count	1.05 (0.92‐1.27)	.40
Fall history (yes vs no)	0.75 (0.35‐1.60)	.45
Practical symptom count	1.05 (0.78‐1.48)	.66
Fears or anxieties (yes vs no)	0.96 (0.73‐1.28)	.75
Fall risk (medication; polypharmacy vs no risk)	1.02 (0.73‐1.42)	.86
Insurance: Medicaid	1.02 (0.31‐2.92)	.97

a IRR: incident rate ratio.

b *ICD*-10: *International Classification of Diseases*, 10th Revision.

c ECOG: Eastern Cooperative Oncology Group.

Higher nutrition symptom counts were associated with greater interruption rates, approaching statistical significance (IRR 1.43, 95% CI 0.99‐2.11; *P*=.051). In contrast, greater physical symptom burden was associated with lower interruption rates (IRR 0.82, 95% CI 0.72‐0.96; *P*=.02).

Marital status and primary cancer site were also significant predictors. Married patients exhibited a lower rate of missed treatments (IRR 0.67, 95% CI: 0.49‐0.92; *P*=.02) compared with unmarried patients. Patients diagnosed with genitourinary cancers had a significantly higher interruption rate (IRR 2.57, 95% CI 1.33‐4.97; *P*=.006), with similar trends observed for head and neck, prostate, and skin cancers.

### XGBoost Modeling

The XGBoost model achieved optimal performance using a maximum tree depth of 4, a learning rate of 0.05, and a subsampling rate of 0.9. Variable importance was assessed using the Gain metric, representing each predictor’s relative contribution to reducing model error (Figure 1).

**Figure 1.**
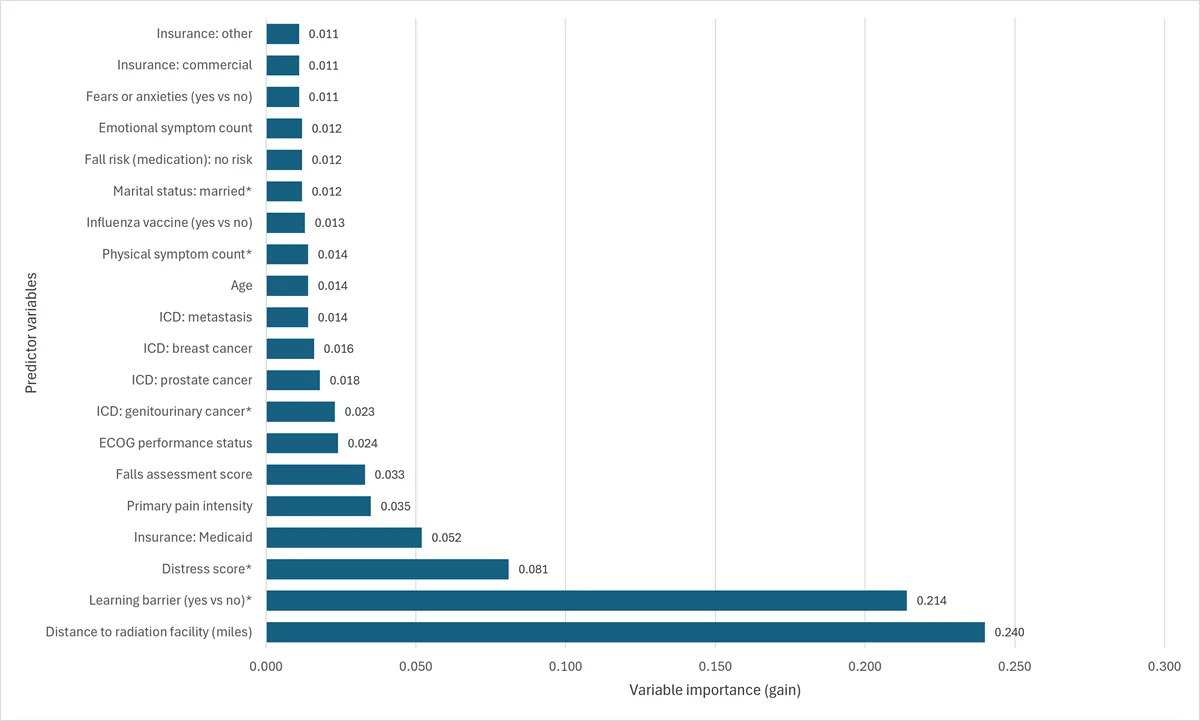
Variable importance in the extreme gradient boosting (XGBoost) model based on the gain metric. Higher values indicate greater relative contribution to prediction accuracy. Variables marked with an asterisk (*) were also statistically significant (*P*<.05) in the negative binomial regression model. ECOG: Eastern Cooperative Oncology Group; *ICD*: *International Classification of Diseases*.

Variables identified with the highest gain were patients’ travel distance to the radiation facility, the presence of learning or communication barriers during treatment, and the total distress score, followed by Medicaid insurance and primary pain intensity. Moderate contributions were observed for fall risk assessment score, ECOG performance status, and genitourinary cancer site.

The Gain metric can favor high-cardinality continuous predictors, such as travel distance, and may produce less stable rankings among correlated features [15]. We therefore interpret the relative ranking of predictors in Figure 1 with this consideration in mind, particularly for travel distance, which may be ranked more highly than its true relative contribution due to this metric’s known bias toward continuous variables with many unique values.

### Predictive Model Comparison

Model performance metrics for the negative binomial and XGBoost models are summarized in Table 3. Both models provided stable and closely aligned predictions of missed radiation treatment days. Each achieved similar predictive accuracy, with XGBoost demonstrating incrementally lower root mean squared error (RMSE=2.19) and mean absolute error (MAE=1.36) vs the negative binomial model (RMSE=2.21; MAE=1.42). Poisson deviance values were also comparable between models, indicating consistent goodness-of-fit across approaches.

**Table 3.** Model performance comparison between negative binomial and extreme gradient boosting (XGBoost) models.

Model	RMSE^a^, mean (SD; 95% CI)	MAE^b^, mean (SD; 95% CI)	Poisson deviance, mean (SD; 95% CI)
XGBoost (Poisson)	2.19 (0.14; 1.95‐2.49)	1.36 (0.05; 1.28‐1.47)	973 (62; 869‐1109)
Negative binomial	2.21 (0.13, 1.99‐2.50)	1.42 (0.05; 1.33‐1.53)	979 (66; 858‐1125)

a RMSE: root mean squared error.

b MAE: mean absolute error.

To place these values in clinical context, we compared both models against a naive baseline predicting the sample mean for all patients, evaluated using the same resampling procedure. The naive baseline achieved a mean RMSE of 2.223 (SD 0.133; 95% CI 1.99‐2.49), only modestly higher than the negative binomial and XGBoost models. This indicates that, while both models offer a measurable improvement in absolute prediction accuracy over a covariate-free estimate, the improvement is limited given the low-count, zero-inflated nature of the outcome.

## Discussion

In this study of patients receiving radiation therapy, we examined how psychological and distress-related symptoms, along with other key clinical and social risk factors, were associated with RTI. Among all studied variables, total distress score emerged as a key determinant, with higher baseline distress levels independently linked to an increased likelihood of missed treatment days across both the negative binomial and XGBoost models. This finding underscores the critical influence of psychological well-being on treatment adherence. Other variables with demonstrated significance in both models, learning or communication barriers during treatment and genitourinary cancer site, highlight complex, multilayered mechanisms leading to RTIs. Strong concordance between traditional regression and machine learning results supports the robustness of identified associations and reinforces the need to incorporate patient-reported data to inform personalized psychosocial and supportive interventions to mitigate preventable interruptions across a multiweek course of daily radiation delivery.

Our study expands upon prior literature demonstrating the influence of emotional distress on radiation therapy adherence. Clover et al [16] found that radiation therapists rated 11% of sessions as disrupted by anxiety at the pretreatment simulation visit, rising to 24% at the first treatment session, with disruptions primarily linked to procedural fears (eg, mask-related anxiety) rather than overall distress. Sreeraman et al [17] found that, among 280 patients with head and neck cancer, poor Karnofsky Performance Status (≤70) and pre-existing psychiatric illness were each significantly associated with a greater likelihood of missing more than 5 treatment days, and Chen et al [18] demonstrated that pretreatment depression independently predicted both prolonged radiation breaks and reduced survival.

Our findings build on this mixed evidence by demonstrating a consistent and independent association between patient-reported distress and RTIs across 2 distinct analytic frameworks. Unlike prior work that relied on diagnostic psychiatric categories or clinician-reported psychological factors [17,19,20], the use of the National Comprehensive Cancer Network (NCCN) Distress Thermometer allowed us to capture real-time, patient-centered indicators of emotional strain. This may explain the stronger and more stable associations observed. Moreover, recent analyses of NCCN Distress Thermometer scores indicate that higher distress correlates with greater acute treatment-related toxicity during radiotherapy, lending additional biological plausibility to distress as a mediator of treatment course deviations [21].

Beyond psychological distress, several additional predictors emerged as important determinants of radiation treatment adherence. Patients with learning or communication barriers consistently showed higher rates of missed sessions, underscoring the role of health literacy and communication effectiveness in sustaining treatment continuity. This aligns with evidence that health system and informational needs are the second most frequently reported unmet supportive care needs among people affected by cancer, behind only psychological and emotional needs, with patients commonly reporting difficulty understanding their diagnosis, treatment, and self-management, as well as challenges navigating and coordinating care [22]. Cancer site was also associated with treatment interruption, with patients with genitourinary and prostate cancers experiencing higher rates of missed treatments. Although prostate cancer is anatomically classified within the genitourinary system, we analyzed it separately because of its high prevalence in our cohort and its distinct treatment patterns. Prior studies have reported higher rates of treatment interruption among patients with head and neck cancer [16,23]; however, head and neck cancer was not among the highest-ranked predictors of treatment interruption in our models. While indicators of functional status such as ECOG performance and fall-risk assessments were not statistically significant, they ranked moderately in the XGBoost model, suggesting that physical limitations may still contribute indirectly to treatment nonadherence. Importantly, evidence from a multiple-baseline intervention study demonstrates that structured pretreatment education delivered by radiation therapists can reduce psychological distress and improve treatment preparedness, supporting the potential of communication-focused interventions to mitigate avoidable treatment disruptions [24]. Taken together, these findings emphasize the value of targeted educational and supportive strategies, particularly for patients with communication challenges or undergoing complex treatment regimens.

Notably, higher physical symptom burden was associated with fewer, rather than more, treatment interruptions in our cohort. This finding contrasts with prior evidence that greater symptom burden is generally associated with worse treatment adherence; for example, each additional moderate-severity symptom during adjuvant endocrine therapy in patients with breast cancer has been associated with 4 to 9 additional missed treatment days [25]. One possible explanation for the divergent direction observed in our study is that patients experiencing greater physical symptoms during radiotherapy may receive closer clinical attention, as prior work has shown that higher symptom severity scores are associated with a greater likelihood of being offered a clinical intervention [26]. Whether this increased clinical contact translates into improved treatment continuity remains untested and should be examined in future prospective work. Alternatively, this association may reflect a form of “healthy adherer” behavior, in which patients more engaged with their overall care are both more likely to accurately report symptoms and more likely to complete treatment as scheduled. Further prospective research incorporating longitudinal symptom tracking is needed to clarify this relationship.

Interestingly, some variables demonstrated high predictive importance in the XGBoost model despite lacking statistical significance in the negative binomial regression. Travel distance to treatment facilities was among the top contributors in the machine learning model, suggesting that geographic accessibility meaningfully influences treatment adherence, even when this effect is not captured well by traditional linear modeling. This could be due to complex, nonlinear relationships, such as threshold effects where adherence declines sharply beyond certain travel distances, or interactions with socioeconomic and logistical factors such as transportation availability or employment constraints. Similarly, insurance coverage and marital status, though not statistically significant, contributed to model accuracy, implying subtle cumulative effects of socioeconomic stability and social support on treatment access. These results highlight the complementary value of machine learning approaches in uncovering interaction and nonlinear effects which conventional regression models may underestimate.

In this study, large language models were incorporated into data preprocessing to improve the usability of unstructured clinical text and enable consistent classification of fields that were otherwise incomplete, inconsistently formatted, or missing structured responses. Model performance varied by variable type. For structured *International Classification of Diseases*, 10th Revision (*ICD*-10) diagnosis codes, GPT-4o achieved an accuracy of 90.8% (95% CI 87.7‐93.9) while for less structured entries such as insurance categorization, accuracy was 81.6% (95% CI 76.7‐86.5). Performance declined further for the 168 distress screening cases in which a nurse had recorded free-text descriptions rather than completing the structured checklist fields; owing to the greater linguistic complexity and variability of this free text, categorization accuracy for these cases was only 48.1% (95% CI 43.0‐53.2). All large language model–generated categorizations were subsequently reviewed and manually refined by our oncology team, and no record was retained in an incorrect category.

Distress was assessed at baseline only, before treatment began, which creates a temporal mismatch with our outcome, as missed treatment days can accumulate over a 4-week to 7-week course of daily fractionated radiation. Baseline distress represents the most actionable time point for early psychosocial intervention and is the only assessment consistently collected within our institution’s standard workflow; however, it may not reflect a patient’s psychological state at the time interruptions occur. Among the 1391 (71.2%) patients with more than one distress screening during treatment (median 2 assessments per patient), 43.7% (n=608) showed a decreased score and 30.3% (n=421) showed an increased score between their first and last measurement, confirming that distress is not static for many patients. Because the number of distress forms did not consistently align with the timing of treatment sessions or interruptions, we were unable to examine whether the distress-RTI association differs between early and later interruptions. Future work incorporating repeated, treatment-aligned distress assessments is needed to clarify this temporal relationship.

Some limitations should be considered when interpreting our findings. First, our retrospective analysis was limited to a single-institutional experience, constraining the generalizability of results to other health systems and patient populations. Also, reliance on electronic health record data introduces the possibility of incomplete or inconsistent documentation, particularly for psychosocial and symptom-related variables. Moreover, area-level socioeconomic indicators derived from census data may not accurately represent individual circumstances. Our outcome measure also does not distinguish patient-initiated absences from provider- or operationally-driven interruptions, as reliably coded reason data were not consistently available, although such interruptions are uncommon in our practice. Finally, while the XGBoost model improved prediction and identified nonlinear effects, it does not infer causality, and variable importance should be interpreted as associative rather than deterministic.

This study integrated clinical, socioeconomic, and patient-reported data to examine how psychological and distress-related symptoms, along with other key clinical and social determinants of health, affect unplanned radiation treatment interruption rates. Both the negative binomial and XGBoost models consistently identified associations of patient-reported distress, learning or communication barriers, and specific cancer types with interrupted treatment. These findings highlight the potential influence of psychological and functional factors on cancer treatment access and a critical need to collect patient-reported hardships to inform social support interventions.

By combining traditional regression and machine learning approaches, this study provides an early but potentially comprehensive understanding of the multifactorial determinants of radiation treatment access. Future work will need to validate reproducible predictive factors across larger, multi-institutional cohorts. Ultimately, such data may inform personalized intervention strategies, such as enhanced psychosocial screening, patient education, and community-centered assistance to support high-quality patient access and treatment efficacy for all patients with cancer requiring radiotherapy.

## Abbreviations

ECOG: Eastern Cooperative Oncology Group
GVIF: generalized variance inflation factor
*ICD*: *International Classification of Diseases*
*ICD*-10: *International Classification of Diseases*, 10th Revision
IRR: incidence rate ratio
MAE: mean absolute error
NCCN: National Comprehensive Cancer Network
RMSE: root mean square error
RTI: radiation treatment interruption
VIF: variance inflation factor
XGBoost: extreme gradient boosting
